# Impaired Air Conditioning within the Nasal Cavity in Flat-Faced *Homo*

**DOI:** 10.1371/journal.pcbi.1004807

**Published:** 2016-03-24

**Authors:** Takeshi Nishimura, Futoshi Mori, Sho Hanida, Kiyoshi Kumahata, Shigeru Ishikawa, Kaouthar Samarat, Takako Miyabe-Nishiwaki, Misato Hayashi, Masaki Tomonaga, Juri Suzuki, Tetsuro Matsuzawa, Teruo Matsuzawa

**Affiliations:** 1 Primate Research Institute, Kyoto University, Inuyama, Aichi, Japan; 2 Institute for Biomedical Sciences, Iwate Medical University, Yahaba, Iwate, Japan; 3 Kanazawa Institute of Technology, Nonoichi, Ishikawa, Japan; 4 RIKEN Advanced Institute for Computational Science, Kobe, Hyogo, Japan; 5 Kanazawa Municipal Hospital, Kanazawa, Ishikawa, Japan; 6 Japan Advanced Institute of Science and Technology, Nomi, Ishikawa, Japan; Iowa State University, UNITED STATES

## Abstract

We are flat-faced hominins with an external nose that protrudes from the face. This feature was derived in the genus *Homo*, along with facial flattening and reorientation to form a high nasal cavity. The nasal passage conditions the inhaled air in terms of temperature and humidity to match the conditions required in the lung, and its anatomical variation is believed to be evolutionarily sensitive to the ambient atmospheric conditions of a given habitat. In this study, we used computational fluid dynamics (CFD) with three-dimensional topology models of the nasal passage under the same simulation conditions, to investigate air-conditioning performance in humans, chimpanzees, and macaques. The CFD simulation showed a horizontal straight flow of inhaled air in chimpanzees and macaques, contrasting with the upward and curved flow in humans. The inhaled air is conditioned poorly in humans compared with nonhuman primates. Virtual modifications to the human external nose topology, in which the nasal vestibule and valve are modified to resemble those of chimpanzees, change the airflow to be horizontal, but have little influence on the air-conditioning performance in humans. These findings suggest that morphological variation of the nasal passage topology was only weakly sensitive to the ambient atmosphere conditions; rather, the high nasal cavity in humans was formed simply by evolutionary facial reorganization in the divergence of *Homo* from the other hominin lineages, impairing the air-conditioning performance. Even though the inhaled air is not adjusted well within the nasal cavity in humans, it can be fully conditioned subsequently in the pharyngeal cavity, which is lengthened in the flat-faced *Homo*. Thus, the air-conditioning faculty in the nasal passages was probably impaired in early *Homo* members, although they have survived successfully under the fluctuating climate of the Plio-Pleistocene, and then they moved “Out of Africa” to explore the more severe climates of Eurasia.

## Author Summary

This is the first investigation of nasal air conditioning in nonhuman hominoids based on computational fluid dynamics with digital topological models of the nasal passage made using medical imaging. Our comparative results of humans, chimpanzees, and macaques show that the inhaled air is conditioned poorly in humans compared with nonhuman primates. We also show that our protruding external nose has little effect on improving air conditioning. The nasal anatomy in *Homo* was weakly sensitive to the ambient atmosphere conditions in evolution, but was formed passively by facial reorganization in this genus. Even though the inhaled air is not adjusted well within the nasal cavity in humans, it can be fully conditioned subsequently in the pharyngeal cavity, which is lengthened in flat-faced *Homo*. Thus, despite an impaired air-conditioning conformation in the nasal passages, *Homo* members must have survived successfully under the fluctuating climate of the Plio-Pleistocene, and then they moved “Out of Africa” in the Early Pleistocene to explore the more severe climates and ecological environments of Eurasia.

## Introduction

A flat, short face is one of the legacies of the genus *Homo* [1, 2]. The facial component remains short and fully below the expanded forehead in this genus, and this contrasts with earlier and contemporary hominins such as the australopithecines, which possessed a long face that protruded away from the brain case in a manner analogous to nonhuman hominids, e.g., chimpanzees [1–3]. Consequently, the external nose protrudes from the face [4], the nasal cavity within the facial cranium is high and quadrangular in a lateral view, and the vertically oriented nasal vestibule is connected close to the floor of the tall nasal cavity in humans [2, 5]. This pattern contrasts with that found in nonhuman primates, which possess a long and triangular nasal cavity, and a horizontally oriented vestibule that is connected vertically with the middle of the cavity [2, 5]. However, the subsequent pharyngeal cavity is much longer in humans than in nonhuman primates [6–10]. The nasal passage, including the nasal vestibule and cavity, conditions the inhaled air, as well as performing other functions such as olfactory sensing, dust filtering, and voice resonance [11, 12]. The pharyngeal cavity also participates in conditioning the air that enters from the nasal cavity [11]. Insufficient conditioning can damage the mucosal tissues in the respiratory system and impair respiratory performance, thereby undermining health and increasing the likelihood of death [11, 12]. Thus, despite the evolutionary modifications in the nasal anatomy in the phyletic divergence of *Homo* from the other hominin lineages, adequate air conditioning must have been maintained, particularly to ensure their successful survival in the severely fluctuating climate of the Pleistocene and their subsequent spread from Africa to Eurasia [1, 13, 14].

In this study, we compared the principles and performance of air conditioning in humans, chimpanzees, and macaques by using a computational fluid dynamics (CFD) model [15] to simulate the airflow and heat and water exchanges over the mucosal surface in the nasal passage. The human CFD model used here simulates the predicted airflow and air-conditioning performance reliable for humans [15]. Three-dimensional models of the nasal passage topology were produced based on tomography scans of the three genera. The models include no paranasal sinuses. We compared the air-conditioning performance in the three genera using the same simulation conditions: heat and water exchange were predicted with a simulation model based on the histological compositions of the mucosal layers and the average surface temperature of the human nasal passage, i.e., 100% relative humidity (% RH) at 34°C (3.34% of the mass fraction of water; % MF) [15]. This means that this study examines the differences in performance that are caused by the anatomical differences of the nasal passage in the three genera, but does not simulate a real performance in nonhuman primates. Whereas similar CFD analyses performed in the same subjects of macaques showed a minor contribution of the maxillary sinus to air conditioning [16], here we examine them again to compare the air-conditioning performance of the three genera using the same simulation conditions. To evaluate the effects of the human external nose, we also produced two virtual topology models: a “no-valve” model where the nasal valve—a narrow slit-like channel between the nasal vestibule and cavity—was removed virtually; and a “horizontal” model where the vertically oriented vestibule was made horizontal, as seen in chimpanzees.

We evaluated the performance among the three species in varied ambient atmospheric conditions, and discuss the evolutionary modifications in air-conditioning performance in the divergence of *Homo* from the other hominin lineages lineage, using nonhuman primates as a model for the latter hominins.

## Materials and Methods

### Ethics statement

This study for animals was performed in strict accordance with the recommendations in the third edition of the Guidelines for the Care and Use of Laboratory Primates at the Primate Research Institute of the Kyoto University (KUPRI), Inuyama, Japan. The protocol was approved by the Animal Welfare and Animal Care Committee at KUPRI (Permit Numbers: 2009–075, 2010–027, 2011–067, and 2012–075). The chimpanzees were anesthetized intramuscularly with 3.5 mg ketamine hydrochloride (Sankyo-Parke-Davis & Co., Inc.) and 0.035 mg medetomidine hydrochloride (Meiji Seika Pharma Co., Ltd., Tokyo, Japan) per kilogram of body weight. The anesthesia was maintained with sevoflurane (Dainippon Sumitomo Pharma Co., Ltd., Osaka, Japan) delivered in oxygen through a precision vaporizer and a rebreathing circuit. The macaques were anesthetized intramuscularly with 2.5 mg ketamine hydrochloride and 0.1 mg medetomidine hydrochloride per kilogram of body weight. Every effort was made to minimize suffering. The daily care and housing facilities strictly conformed to the recommendations in the third edition of the Guidelines for the Care and Use of Laboratory Primates at the KUPRI. To ensure the animals' health and welfare, their general appearance was daily monitored and recorded, along with their daily food and fluid intake.

This study for humans was performed in strict accordance with the recommendations in the Declaration of Helsinki, Ethical Principle of Medical Research Involving Human Subjects prepared by the World Medical Association. All subjects gave an informed consent. The protocol was approved by the Human Research Ethics Committee of KUPRI (Permit Number: H2011-06).

### Subjects and tomographic scanning

Ten non-human primates—four chimpanzees, *Pan troglodytes* [17], four Japanese macaques, *Macaca fuscata*, and two Rhesus macaques, *M*. *mulatta*—which were reared at KUPRI, Inuyama, Japan, were scanned using a computed tomography scanner (Asteion Premium 4, Toshiba Medical Systems Co., Otawara, Japan) at the KUPRI (S1 Table). The two species of macaques are here regarded as subjects of a same genus *Macaca* along with a genus *Pan*. All of the CT scans obtained in this study came from subjects without any history of surgery and had few abnormal traits in their heads, and few artifacts distorted the images of the nasal region. The scans were registered under PRICT # (S1 Table) and are available via the website of the Digital Morphology Museum of KUPRI (dmm.pri.kyoto-u.ac.jp/archive/). The scans of macaques used here were also used for another CFD study [16].

Six human volunteers were scanned with a magnetic resonance imaging scanner (Magnetom Verio, Siemens AG, Munich, Germany) at the Brain Activity Imaging Center, ATR-Promotions, Seika, Japan (S1 Table). Among them, the following CFD simulation analyses used the scans of the five subjects who had no surgery, few abnormal traits, and few artifacts distorting the images in the nasal region.

### Analyses of computational fluid dynamics

The CFD simulations with heat and water exchange were performed as described [15, 18]. The physiological model used here, developed based on the previous models [18], reflects human respiratory physiology and histology, including latent heat, to simulate well real performances of the airflow, heat and water exchanges for humans [15]. The following procedures of the nasal topological models and CFD analyses are the same as those used by Hanida et al. [15], excluding those for generating the virtual topological models. It is slightly different from the study of Mori et al. [16] using the same macaques, in simulation model and boundary conditions.

### Models of the nasal passage anatomy

The voxel data of the nasal passage anatomy were reconstructed from computed tomography scans for nonhuman primates and magnetic resonance imaging scans for human volunteers. The black area representing the air filling the nasal passage was extracted first by using a threshold of brightness with Avizo 7 (FEI), and then the voxel data were reconstructed. After converting the voxel data to STereo Lithography (STL) data, these were modified into data representing the smooth surface using Magics 9.5 (Materialize Inc., Leuven, Belgium). Finally, a tetrahedron mesh with the mesh size of Δx = 2.10 to 3.65×10^−4^ mm, depending on the size of the subjects, was generated from the modified STL data using Gambit 2.4 (ANSYS Inc., Canonsburg, PA, USA; S1 Table). The computational meshes had 2.66 to 3.70 million tetrahedral cells (S1 Table). The present solutions are evaluated independently to the mesh size: there were few differences between the solutions by the present mesh size and the minimum mesh size (Δx = 2.00 to 3.30×10^−4^ mm, depending on the size of the subjects) in each subject; i.e., up to 1.6% of the flow velocity for the same frontal contour.

### Virtual models of the nasal passage anatomy

**Fig 1:**
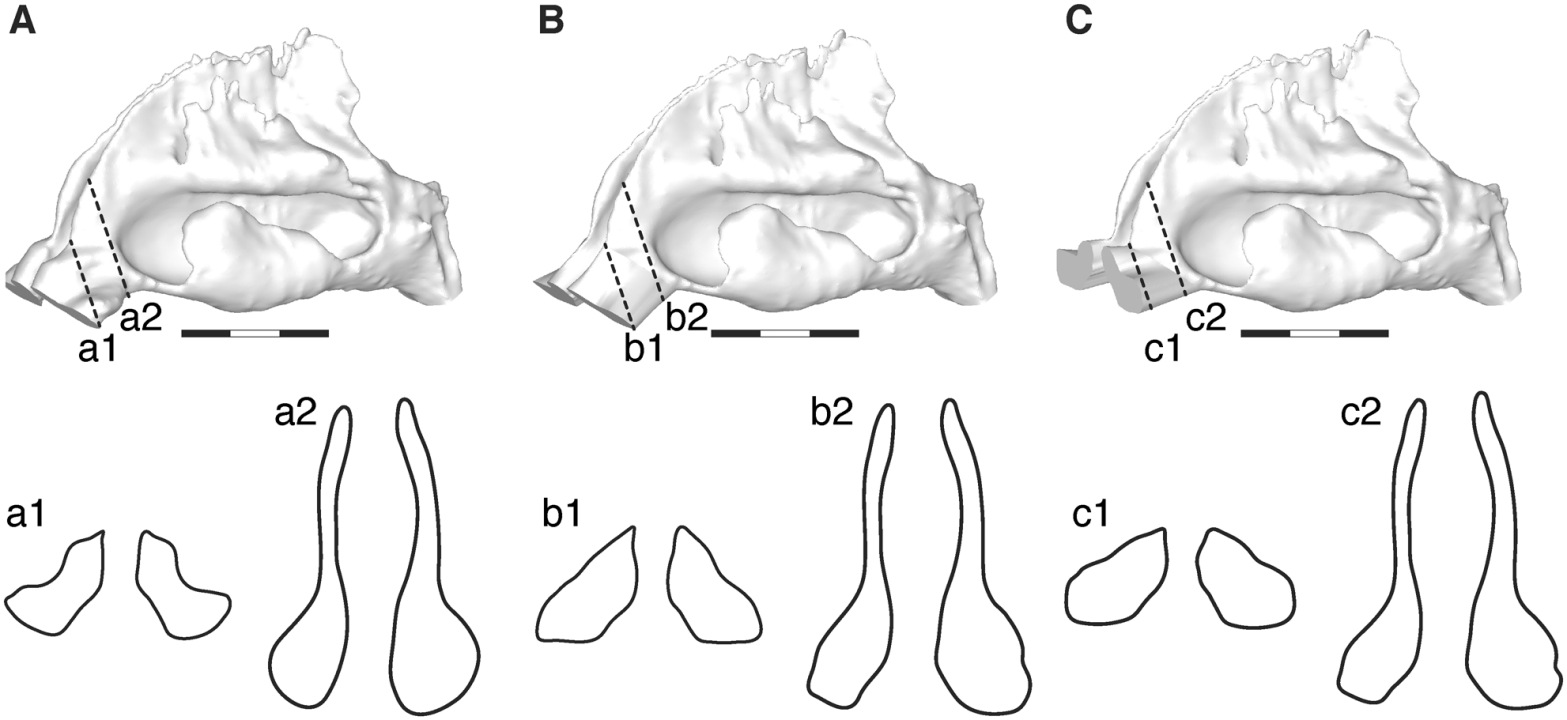
Virtual nasal topology models. (A) Normal, (B) no-valve, and (C) horizontal models. Human volunteer 2. The two contours in the dotted lines show the geometry in the vestibular region, respectively.

The no-valve and horizontal nasal vestibular topology models were generated from the original smoothed STL data using Rhinoceros (AppliCraft Co., Ltd, Tokyo, Japan). We defined the basal plane for making the modifications as almost parallel to the narrow channel between the nasal vestibule and the nasal cavity, which approximately corresponded to the nasal valve (Fig 1A). Using the loft function in Rhinoceros, we generated a straight surface between the basal plane to the nostril on each side, thereby removing the effects of the nasal valve (i.e., the no-valve model; Fig 1B). Next, we tilted the modified vestibule with its straight surface upward, thereby making its lower surface horizontal relative to the floor of the nasal cavity, as seen in the vestibule of chimpanzees (i.e., the horizontal model; Fig 1C; tilting angle: vol.1, 52 degrees; vol.2, 32 degrees; vol.3, 40 degrees; vol.5, 46 degrees, vol.6, 36 degrees).

### Simulation model of inhaled airflow

We performed steady-state analyses to examine the airflow, where the turbulence model was not employed. A steady simulation is reasonable under a normal breathing frequency and flow rate in the resting stage in humans [15, 18–24]. The maximum Reynolds numbers ranges from 135 to 1264 at the position of pharyngeal cavity where its cross-sectional area was measured in the subjects use here, by calculated with estimates of the inhaled air velocity in the resting stage (see the subsection **Boundary conditions for calculation**). These values are lower than the critical Re value of 2300 denoting the transition between laminar to turbulent flow, and much lower than the Re of 5000 which is considered to be completely turbulent [19, 21, 24]. The nasal flow is regarded as being mostly laminar in the resting stage of the subject used here. The Strouhal number value for the system is less than 0.25 [24]. Moreover, the Womersley number for human breathing is small, thereby indicating that any inertial effects on the flow pattern may be regarded as negligible [24, 25]. We used the CFD simulation model developed by Hanida et al. [15] to model an incompressible, viscid, laminar airflow in the nasal cavity with heat and water transport. The equations were solved using the fluid simulation software FLUENT 6.3 (ANSYS Inc., Canonburg, USA).

The simulation was governed by the Navier–Stokes equation—the conservation of momentum Eq (1)—by the equation of conservation of mass Eq (2), by the transport equation of energy Eq (3), and by the transport equation for the mass fraction of water Eq (4).

$$\rho \left\{\frac{\partial u}{\partial t}+\left(u\cdot \nabla \right)u\right\}=-\nabla p+\mu \nabla^{2}u$$ (1)

∇⋅u=0 (2)

$$\rho Cp\left\{\frac{\partial T}{\partial t}+\left(u\cdot \nabla \right)T\right\}=K\nabla^{2}T$$ (3)

$$\frac{\partial F}{\partial t}+\left(u\cdot \nabla \right)F=D\nabla^{2}F$$ (4)

Here *t*, **u**, *p*, *ρ*, *v*, *K*, *T*, *Cp*, *F*, and *D* denote time, velocity, pressure, density, kinematic viscosity, thermal conductivity, temperature, specific heat, mass fraction of water, and mass diffusion coefficient, respectively. We regarded a solution as being of steady-state, after *T* (time) advanced substantially: we repeated the steps of calculation sufficiently until the values of system parameters reach the criterions of convergence, i.e, continuity, X,Y,Z-velocity, energy, and H_2_O reach 1×10^−4^, 1×10^−5^, 1×10^−7^, and 1×10^−4^, respectively, by the FLUENT 6.3.

### Wall model for heat and water exchange

**Fig 2:**
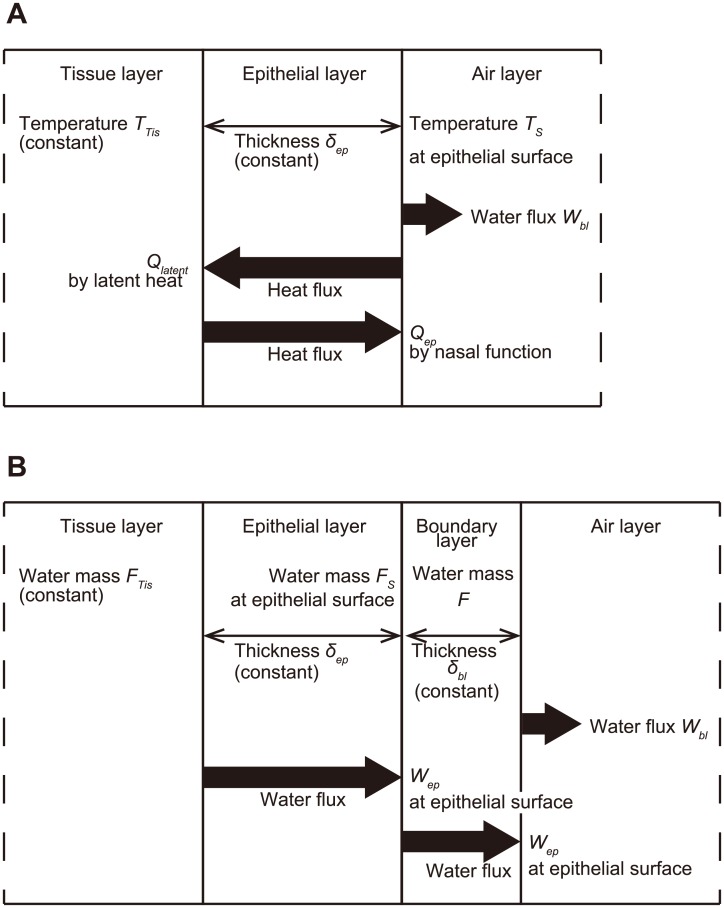
Wall models of the nasal passage. (A) Model for heat exchange. *Q*_*ep*_, *Q*_*latent*_, and *W*_*bl*_ indicate the heat flux of the nasal wall’s function, the heat flux of latent heat, and the water flux from the boundary layers in (B). *T*_*S*_, *T*_*Tis*_, and *δ*_*ep*_ are the temperature of the surface, the tissue layer temperature, and the epithelial layer thickness, respectively. (B) Model for water exchange. *W*_*bl*_ and *W*_*ep*_ are the water flux from the boundary layer and the water flux from the tissue layer. *F*, *F*_*S*_, *F*_*Tis*_, and *δ*_*bl*_ are the water fraction in the boundary layer, the water fraction on the epithelial surface, the water fraction on the tissue layer, and the boundary layer thickness, respectively.

The wall of the nasal passage was modeled by the tissue and epithelial layers (Fig 2) to simulate the exchange of heat and water from the vascular layer to the air via the mucous membrane [15]. The mucous membrane includes the membrane epithelia, nasal glands, blood vessels, and capillary blood vessels [26], and thus it varies in thickness between 0.3 and 5 mm according to location in humans. The present model was composed of a smooth surface and a constant thickness of 0.2 mm in the nasal vestibular region and 0.5 mm in the other region, which was specified to simulate the actual performance in humans, after Hanida et al. [15]. Thus, the predicted air-conditioning performance may be slightly worse than that actually found in smaller-bodied chimpanzees and macaques having thinner mucosal layers. The optimum values of the temperature and humidity of the inhaled air were calculated by setting a boundary condition representing the heat and water exchange on the surface of the epithelial layer.

### Simulation model of heat exchange

Fig 2A illustrates the present simulation model for heat exchange [15, 18]. Heat is transferred between the air and the tissue layer via the epithelial layer. The heat transport of *Q*_*ep*_ from the tissue side is determined by Eq (5). The latent heat of *Q*_*latent*_ is calculated from Eq (6), where *L* and *W*_*bl*_ denote the specific latent heat and water flux from the surface of the epithelial layer, respectively. *L* is defined by Eq (7), which was calculated by cubic fitting to the data reported by Rogers and Yau [27]. The total heat transport, *Q*_*total*_, is defined by Eq (8) as a flux boundary condition for the energy Eq (3).

$$Q_{ep}=K_{ep}\frac{T_{S}-T_{Tis}}{\delta_{ep}}$$ (5)

$$Q_{latent}=-LW_{bl}$$ (6)

$$L=2500.79-0.00000614342\times T_{S}^{3}+0.00158927\times T_{S}^{2}-2.36418\times T_{S}$$ (7)

$$Q_{total}=Q_{ep}+Q_{latent}=K_{ep}\frac{T_{S}-T_{Tis}}{\delta_{ep}}-LW_{bl}$$ (8)

*T*_*S*_, *T*_*Tis*_, *K*_*ep*_ and *δ*_*ep*_ denote the temperature of the surface, the tissue layer temperature, the thermal conductivity of the epithelial layer and the epithelial layer thickness, respectively. *T*_*Tis*_ is constant, set here at 34°C, the temperature commonly measured in the human nasal region. The predicted air-conditioning performance is slightly inferior compared with that in reality for chimpanzees and macaques having higher body temperature. Nevertheless, this value is commonly used here for simulation both in humans and non-human primates, to examine the differences of air-conditioning performance which are caused by the morphological differences in nasal passage anatomy between them. The thermal conductivity of the mucous membrane *K*_*ep*_ is 0.6 W/mK that is thermal conductivity of water [28], because the liquid mucous membrane is assumed in the model used here [15, 18]. *T*_*S*_ is determined by *Q*_*total*_, which comprises *Q*_*ep*_ and *Q*_*latent*_.

### Simulation model of water exchange

Fig 2B illustrates the wall model, implemented with a boundary layer to define the boundary condition of species transport for water exchange [15,18]. The model is based on Fick’s law in that the flux diffusion is proportional to the concentration gradient of water. Here, we used the Dirichlet-type boundary condition (i.e., fixed transport) in the FLUENT software. The two-film theory was used here to evaluate the mass of species transport between a liquid phase and a gas phase across a boundary. The thickness of the boundary layer was set at 0.5 mm [15]. *W*_*bl*_ is the water flux from the boundary layer, which is determined from Eq (9), and this was used to calculate the latent heat of Eqs (6) and (8). *W*_*ep*_ is the water flux from the tissue layer, which is determined from Eq (10).

$$W_{bl}=D_{bl}\frac{F-F_{S}}{\delta_{bl}}$$ (9)

$$W_{ep}=D_{ep}\frac{F_{S}-F_{Tis}}{\delta_{ep}}$$ (10)

Here *F*, *F*_*S*_, *F*_*Tis*_, *δ*_*bl*_, *δ*_*ep*_, *D*_*bl*_, and *D*_*ep*_ denote the water fraction in the boundary layer, the water fraction on the epithelial surface, the water fraction on the tissue layer, the boundary layer thickness, the epithelial layer thickness, mass diffusion coefficient of the boundary layer, and mass diffusion coefficient of the epithelial layer, respectively. *D*_*bl*_ and *D*_*ep*_ are 3.0 × 10^−5^ m^2^/s and 2.6 × 10^−5^ m^2^/s, respectively [29]. *F*_*Tis*_ is 3.34% of the water mass fraction in 100% of the relative humidity at 34°C. It is noted that diffusion in the boundary layer is greater than that of the membrane layers [15]. The water flux is transported from the tissue through the epithelial and boundary layers to the air. Simultaneously solving Eqs (9) and (10) for *F*_*S*_ gives Eq (11). The temperature is not dominant in Eqs (9–11), and the water transport is not regarded as being dependent on a temperature in this model. To enable mass flux of species transport, *F*_*S*_ was fixed as the boundary condition for water exchange. This boundary condition was implemented as a user-defined function in FLUENT software.

$$F_{S}=\frac{\left(\frac{D_{ep}}{\delta_{ep}}\right)F_{Tis}-\left(\frac{D_{bl}}{\delta_{bl}}\right)F}{\left(\frac{D_{ep}}{\delta_{ep}}\right)+\left(\frac{D_{bl}}{\delta_{bl}}\right)}$$ (11)

Note that the nasal vestibule is covered with epidermis, where water is not exchanged [15].

### Boundary conditions for calculation

The external nostril was modeled as a free inlet, and no-slip boundary conditions were applied at the walls, while the outward velocity was assigned at the pharynx [15].

The time-averaged velocity of the inhaled air at the pharynx was calculated based on estimates of the resting tidal volume and the respiratory rate, as well as the measurement of the cross-sectional area of the pharyngeal region at a given position for each subject (S1 Table). The cross-sectional area was calculated at a given location in the pharynx based on the CT scans using Magics software.

The resting tidal volume was estimated by Eq (12) [30].

$$TV=7.69BW^{1.04}$$ (12)

Here, *TV* and *BW* denote the estimate of the resting tidal volume (ml) and measured body weight (kg), respectively.

The resting respiratory rate was estimated by Eq (13) [31].

$$f=0.84BW^{-0.26}$$ (13)

Here, *f* denotes the estimate of the respiratory rate (breaths/second, Hz).

Finally, the time-averaged velocity was calculated by Eq (14).

$$FV=\frac{2f\times TV}{CA}$$ (14)

Here, *FV* and *CA* denote the time-averaged flow velocity (m/s) and the measured cross-sectional area at a given location of the pharynx (mm^2^), respectively.

The CFD simulations were performed in three ambient atmospheric air conditions: cold–dry, 10% RH at 5°C (0.05% MF); hot–dry, 5% RH at 40°C (0.23% MF); and warm–wet, 60% RH at 30°C (1.58% MF).

### Visualizations

The resulting spatial pattern of the vector quantity representing the velocity and direction of the airflow is illustrated using streamlines in different colors that was computed from the points on the plane of external nostrils [15, 18]. The number of streamlines is decided dependent on the area of the plane of nostrils, and it reflects the relative airflow volume for a given subject, allowing us to examine where the air mainly flows. Those of the scalar quantity representing the temperature and water vapor volume are illustrated using contours in different colors [15, 18].

## Results

**Fig 3:**
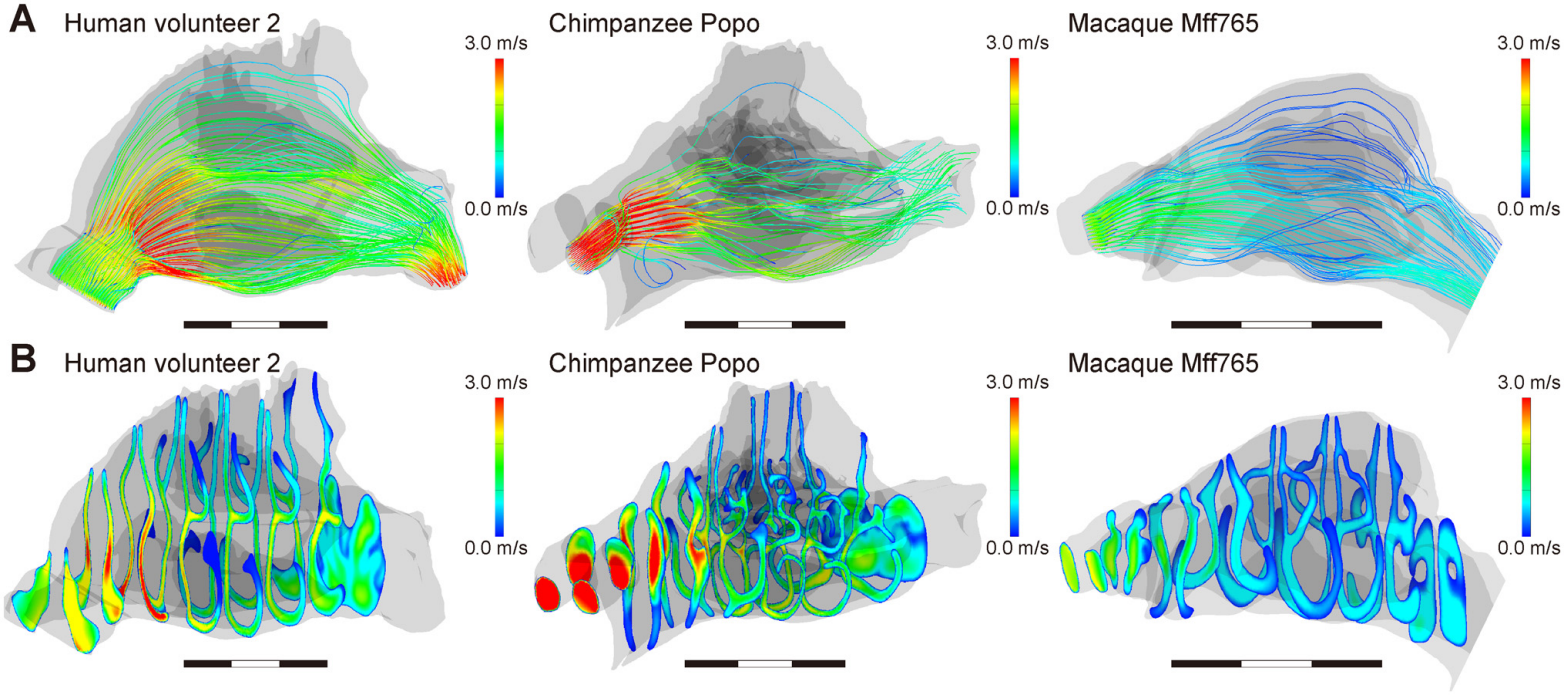
Airflow and flow velocity in the nasal passage. (A) Human volunteer 2, (B) chimpanzee Popo, and (C) macaque Mff765. The streamlines (upper) and the contours (bottom) indicate the airflow direction and velocity distributions through the nasal passage, respectively. The number of streamlines reflects the relative airflow volume for a given subject.

Our simulations of CFD showed that the airflow direction in the nasal cavities of chimpanzees and macaques differs in some key regions from that in humans. In humans, the inhaled air flows upward along the ascending vestibular region into the nasal cavity and downward to the oropharynx (Fig 3A and S1 Fig), whereas in chimpanzees and macaques the air flows straight from the horizontal vestibule, through the nasal cavity, and out to the oropharynx (Fig 3A and S2 Fig). Our predictions for humans agree with many studies of different humans with variable external nose and nostril morphologies [19, 23, 32–38]. However, irrespective of the differences in the flow direction, the air passes through the mid-medial to the inferior region in greater volume and velocity compared with the peripheral and superior regions of the nasal cavity in all three genera in a similar manner (Fig 3 and S1 and S2 Figs). Although the location differs slightly, this major flow passage was also determined in many previous experimental and CFD simulation studies in humans [19, 20, 23, 32–38] and macaques [39]. Thus, the major flow passage through the nasal cavity in humans is almost the same as in chimpanzees and macaques, although humans have an upward and curved airflow.

**Fig 4:**
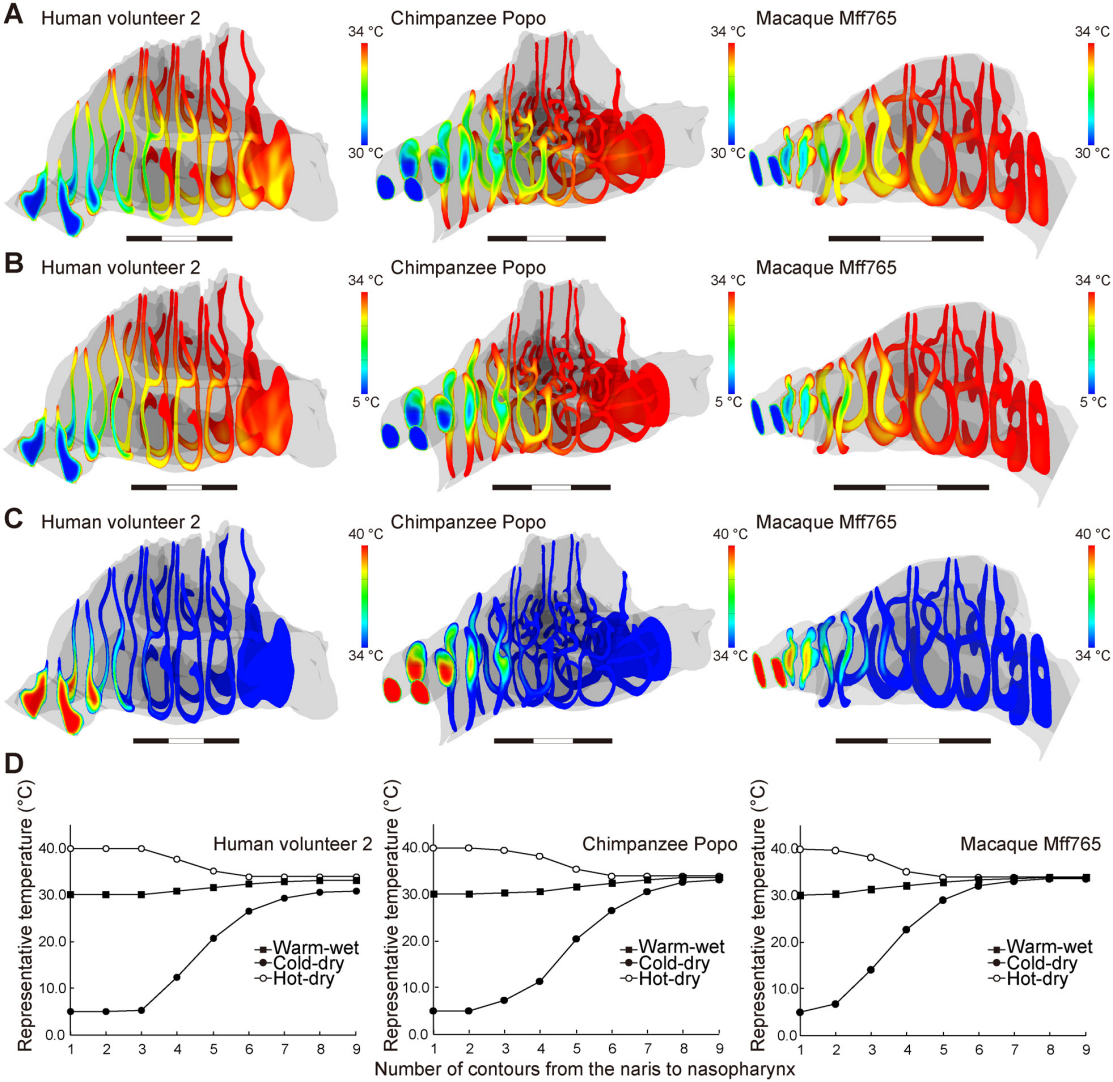
Distribution of temperature in the nasal passage. (A) Warm and wet; (B) cold and dry; and (C) hot and dry conditions. The contours represent the distribution of temperature at each level from the nares to the nasopharynx. (D) The lowest temperatures in each contour were in the warm/wet and cold/dry conditions, and the highest temperatures were in the hot/dry conditions. The values indicate the performance when adjusting the temperature toward 34°C.

**Fig 5:**
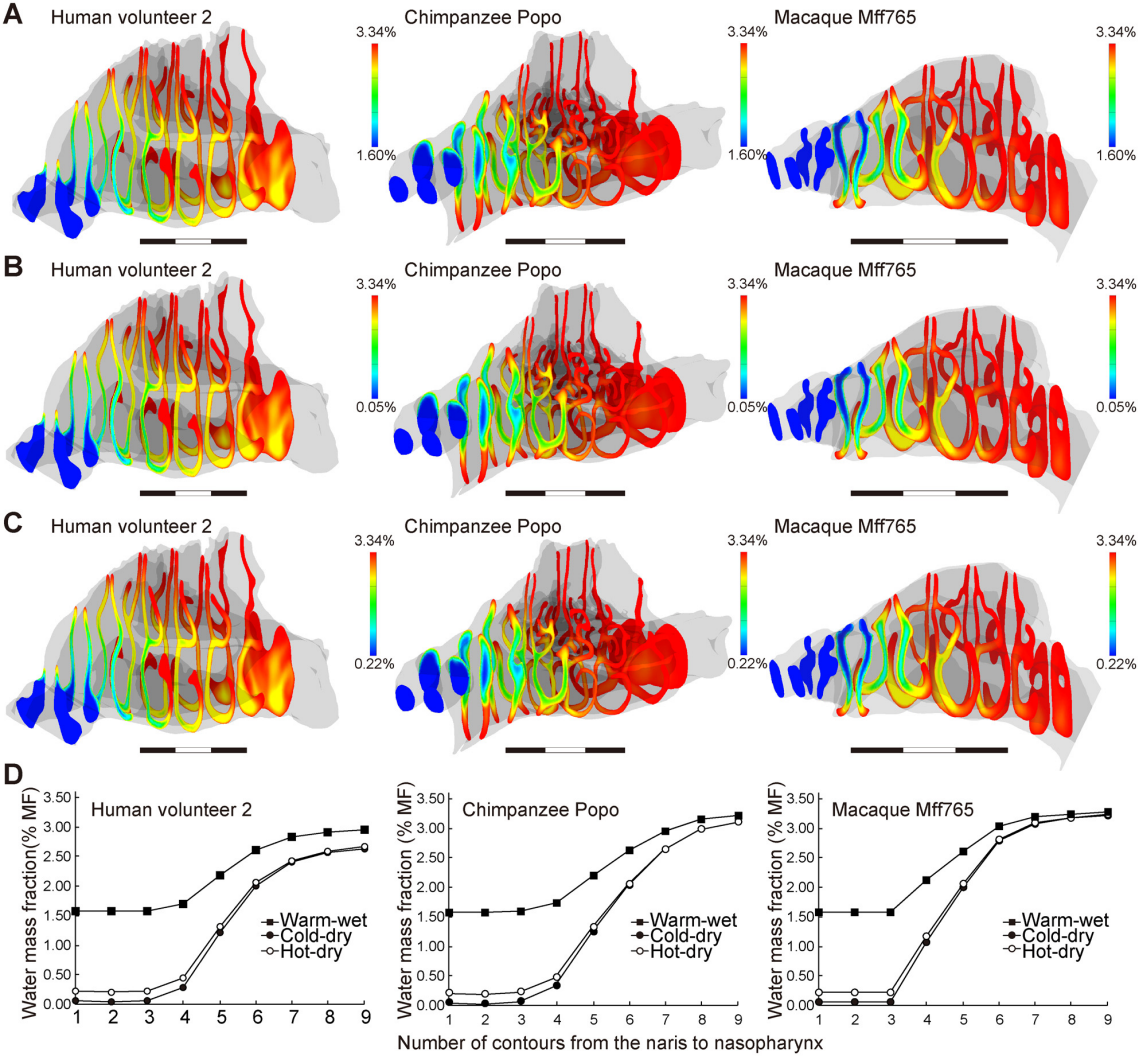
Distribution of the water mass fraction in the nasal passage. (A) Warm and wet; (B) cold and dry; and (C) hot and dry conditions. The contours represent the distribution of the water mass fraction at each level from the nares to the nasopharynx. (D) The lowest values of the water mass fraction were measured at each level and indicate the performance when modifying the water vapor in the inhaled air toward the optimum mass fraction of water: 3.34%.

In our simulations, the inhaled air was conditioned well in chimpanzees and macaques, even in severe ambient conditions (Figs 4 and 5; Table 1), where the inhaled air was adjusted to approximately 34°C and saturated to almost 100% RH before reaching the nasopharyngeal region, i.e, the most posterior frontal contour (Figs 4 and 5; Table 1). However, the air-conditioning performance was lower in humans according to our CFD model. Thus, the air was not adjusted fully in the two dry conditions in that air with <80% of the required water content at 34°C remained in the nasopharyngeal region (Figs 4 and 5; Table 1). The cold and dry air was not well adjusted in terms of temperature so that air at <30°C also remained in the nasopharyngeal region (see the most posterior contour on Figs 4B, 4D, 5B and 5D; Table 1), whereas the hot and dry air was adjusted well to 34°C (Figs 4B, 4D, 5B and 5D; Table 1). The warm and wet air was adjusted well to over 32°C at the nasopharyngeal level, but it was not fully adjusted in terms of humidity: air with <90% of the required water content at 34°C remained there (Figs 4A, 4D, 5A and 5D; Table 1). Nevertheless, such worse performances probably reflect the air-conditioning performance in healthy humans well. In fact, the air is often only conditioned up to 30°C or to 80–90% RH in the nasopharyngeal region of humans, even in comfortable ambient conditions [40–42]. Thus, inhaled air is not well conditioned in the nasal cavity of humans compared with nonhuman primates.

**Table 1:** Temperature, water mass fraction, and relative humidity at the nasopharyngeal level.

Species	Subjects	Warm and wet		Cold and dry		Hot and dry	
		T (°C)	MF (RH34) (%)	T (°C)	MF (RH34) (%)	T (°C)	MF (RH34) (%)
**Humans, *Homo sapiens***	**Volunteer 1**	**32.6**	**2.68 (80.3)**	**26.7**	**2.12 (63.4)**	**34.2**	**2.18 (65.2)**
	**Volunteer 2**	**33.1**	**2.96 (88.5)**	**30.9**	**2.64 (78.9)**	**34.0**	**2.66 (79.6)**
	**Volunteer 3**	**33.6**	**3.14 (94.1)**	**32.8**	**2.98 (89.2)**	**34.0**	**2.99 (89.6)**
	**Volunteer 5**	**32.7**	**2.75 (88.2)**	**27.5**	**2.24 (67.1)**	**34.0**	**2.29 (68.7)**
	**Volunteer 6**	**32.9**	**2.82 (84.3)**	**28.6**	**2.37 (71.1)**	**34.0**	**2.42 (72.3)**
	**Average**	**33.0**	**2.87 (85.9)**	**29.3**	**2.47 (74.0)**	**34.0**	**2.51 (75.1)**
**Chimpanzees, *Pan troglodytes***	**Mari**	**33.7**	**3.22 (96.4)**	**33.2**	**3.13 (93.7)**	**34.0**	**3.13 (93.7)**
	**Pendesa**	**33.8**	**3.24 (97.1)**	**33.3**	**3.16 (94.8)**	**34.0**	**3.17 (94.8)**
	**Popo**	**33.7**	**3.21 (96.2)**	**33.1**	**3.11 (93.2)**	**34.0**	**3.11 (93.2)**
	**Reiko**	**33.6**	**3.19 (95.5)**	**32.8**	**3.07 (92.1)**	**34.0**	**3.08 (92.1)**
	**Average**	**33.7**	**3.21 (96.3)**	**33.1**	**3.12 (93.3)**	**34.0**	**3.08 (93.5)**
**Japanese macaques, *Macaca fuscata***	**Mff765**	**33.9**	**3.27 (98.0)**	**33.7**	**3.22 (96.5)**	**34.0**	**3.23 (96.6)**
	**Mff963**	**33.8**	**3.21 (96.1)**	**33.3**	**3.10 (92.9)**	**34.0**	**3.11 (93.2)**
	**Mff1859**	**34.0**	**3.33 (99.8)**	**34.0**	**3.33 (99.7)**	**34.0**	**3.33 (99.7)**
	**Mff2115**	**34.0**	**3.33 (99.6)**	**33.9**	**3.32 (99.4)**	**34.0**	**3.32 (99.4)**
**Rhesus macaques, *Macaca mulatta***	**Mm1701**	**34.0**	**3.34 (99.9)**	**34.0**	**3.34 (99.9)**	**34.0**	**3.34 (99.9)**
	**Mm1715**	**34.0**	**3.34 (99.9)**	**34.0**	**3.34 (99.9)**	**34.0**	**3.34 (99.9)**
**Macaques, *Macaca***	**Average**	**34.0**	**3.30 (98.9)**	**33.8**	**3.28 (98.1)**	**34.0**	**3.28 (98.2)**

T, the lowest values of temperature in the nasopharyngeal level (the most posterior contour in figures for a given subject) were for the warm-humid and cold-dry conditions, and the highest value was for the hot-dry condition; MF, the lowest values of the water mass fraction in the nasopharyngeal level; RH34, percentage of the water mass fraction to 3.34%, the water mass fraction at which the air of 34°C is fully saturated.

**Fig 6:**
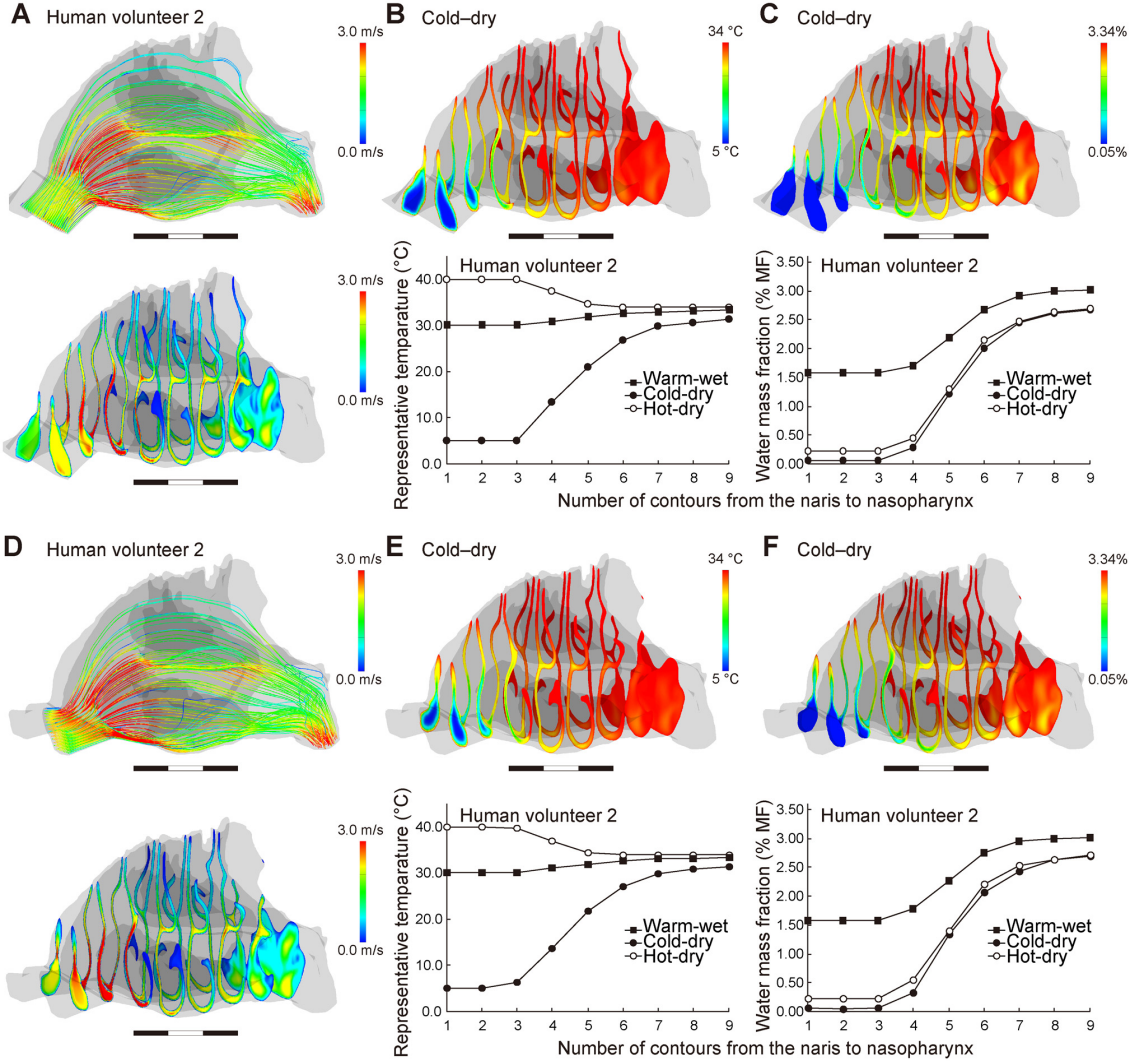
Airflow, temperature, and water mass fraction in the nasal passages of the virtual models. Human volunteer 2. (A–C) No-valve model and (D–F) horizontal models. (A, D) Airflow, the streamlines (upper) and the contours (bottom), (B, E) temperature, the contours (upper) and the representative values in each contour (bottom), and (C, F) water mass, the contours (upper) and the lowest values in each contour (bottom). See Figs 3–5 for details.

The no-valve model resulted in a few changes in the airflow direction and air-conditioning performance for each human subject (Fig 6A–6C; S3 Fig; Table 2). The normal model exhibited a fast and diffusive flow through the nasal valve in all three genera (Fig 3, S3 Fig), but this flow was not found in the human no-valve model (Fig 6A; S3 Fig). In the horizontal model, the airflow direction was changed to be slightly horizontal and straight, as seen in chimpanzees, but it had only a minor effect on the air-conditioning performance: the temperature and humidity at the nasopharyngeal level did not differ from those in the normal and no-valve models for each subject (Fig 6D–6F; S3 Fig; Table 2). Thus, the vertically oriented vestibule contributes to the upward airflow, but the topology of the nasal valve and vestibule makes little contribution to improving the air-conditioning performance in humans.

**Table 2:** Temperature, water mass fraction, and relative humidity at the nasopharyngeal level for the virtual models.

Models	Subjects	Warm and wet		Cold and dry		Hot and dry	
		T (°C)	MF (RH34) (%)	T (°C)	MF (RH34) (%)	T (°C)	MF (RH34) (%)
**No-valve**	**Volunteer 1**	**32.6**	**2.71 (81.3)**	**26.9**	**2.18 (65.2)**	**34.2**	**2.24 (67.0)**
	**Volunteer 2**	**33.3**	**3.01 (90.2)**	**31.4**	**2.67 (79.9)**	**31.5**	**2.69 (80.7)**
	**Volunteer 3**	**33.6**	**3.21 (96.2)**	**33.0**	**3.01 (90.2)**	**34.0**	**3.02 (90.4)**
	**Volunteer 5**	**32.6**	**2.71 (88.1)**	**27.1**	**2.18 (65.2)**	**34.1**	**2.24 (66.9)**
	**Volunteer 6**	**32.8**	**2.80 (83.8)**	**28.2**	**2.34 (70.1)**	**34.0**	**2.38 (71.4)**
	**Average**	**33.0**	**2.89 (86.5)**	**29.3**	**2.48 (74.1)**	**33.6**	**2.51 (75.3)**
**Horizontal**	**Volunteer 1**	**32.4**	**2.56 (76.5)**	**26.2**	**1.88 (56.4)**	**34.2**	**1.95 (58.5)**
	**Volunteer 2**	**33.3**	**3.02 (90.5)**	**31.5**	**2.68 (80.4)**	**34.0**	**2.71 (81.2)**
	**Volunteer 3**	**33.6**	**3.20 (95.9)**	**33.1**	**3.04 (90.9)**	**34.0**	**3.10 (92.8)**
	**Volunteer 5**	**32.5**	**2.69 (80.5)**	**27.0**	**2.14 (64.0)**	**34.0**	**2.19 (65.6)**
	**Volunteer 6**	**32.8**	**2.78 (83.3)**	**28.2**	**2.31 (69.2)**	**34.0**	**2.35 (70.4)**
	**Average**	**33.0**	**2.85 (85.3)**	**29.2**	**2.41 (72.2)**	**34.0**	**2.46 (73.7)**

T, the lowest values of temperatures in the nasopharyngeal level (the most posterior contour in figures for a given subject) were for the warm-humid and cold-dry conditions, and the highest value was for the hot-dry condition; MF, the lowest values of the water mass fraction in the nasopharyngeal level; RH34, percentage of the water mass fraction to 3.34%, the water mass fraction at which the air of 34°C is fully saturated.

## Discussion

In this CFD study, we used the same simulation conditions to compare the air-conditioning performance in humans and non-human primates. It should be noted that MRI scans were used for reconstructing the nasal passage models in humans, while CT scans were used in nonhuman primates. A same type of tissue is resolved differently between the two modalities. In a precise sense, mixing CT and MRI scans potentially alters the thickness of the airway and flow velocities for a same subject. Nevertheless, the present results using MRI scans also show an airflow pattern, velocities, and air-conditioning performance that are similar to those by a same CFD model using a CT-based model in humans [15]. Such mixing is available for gross comparisons [43], as like the present gross comparison of humans and nonhuman primates. Thus, our study provides evidence supporting for the view that the air-conditioning performance within the nasal cavity is less effective in humans than in nonhuman primates.

The nasal topology is often regarded as being evolutionarily sensitive to the ambient atmospheric conditions of a habitat such as temperature and humidity for a given clade [45–48]. In fact, the inhaled air is already fully conditioned in the anterior region of the nasal cavity in chimpanzees and macaques. This finding does not contradict another CFD study using macaques and savannah monkeys, despite using slightly different CFD methods and conditions [16]. These findings mean that the morphology of the nasal cavity can accept some morphological evolutionary modifications that might impair air-conditioning in nonhuman primates. The earlier hominins other than the genus *Homo* have a nasal passage in a manner analogous to chimpanzees rather than humans, suggesting that they probably show the effective air-conditioning performance as seen in non-human primates. However, the characteristic facial reorganization in *Homo* has precluded the developmental elongation of the oral and nasal cavities [7–10] and has impaired their air-conditioning performance. The nasal cavity anatomy is believed to vary with an advantage to the climate conditions of the habitat of a given population in modern humans [47, 48], but the present finding supports the idea that morphological modification in the nasal region is only a weak evolutionary response to air-conditioning needs in the divergence of *Homo* from the other hominin lineages. Rather, the nasal region is regarded just as a buffering module for facial reorganization, in contrast with other modules, including the jaw, eye, and braincase, which have been modified yet have maintained their independent functions. Thus, human nasal topology was probably modified passively by evolutionary facial reorganization in early *Homo*, and such an evolutionary modification was not prevented by impaired the air-conditioning performance.

The unique external nose in *Homo* is believed to confer some functional advantages during air conditioning, such as retaining the water vapor from expired air [4] or generating a vortex airflow with inhaled air to improve air conditioning [2, 44]. The nasal vestibule within the external nose is coated with epidermis, including vestibular hairs, and it only exchanges heat with the air, which means that the nasal vestibule itself makes a limited contribution to the air conditioning that occurs within it. Our study confirmed that the nasal valve also has little effect on the air-conditioning performance in humans. In fact, the topography of the nasal valve has specific effects on the local airflow pattern within the nasal cavity such as vortices in the superior meatus [34], but there are limited effects on the gross airflow pattern, including turbulence [34, 44]. Further, our study also showed that the vertically oriented vestibule makes a major contribution to the generation of an upward airflow in the nasal cavity in humans, but the inhaled air is still well conditioned mainly in the mid-medial to inferior regions of the nasal cavity as seen in intact humans and non-human primates. Although the location differs slightly, such a major flow passage was confirmed in humans with variable external nose and nostril morphologies in modern humans [38]. Irrespectively of varied nostrils, the vertically oriented vestibule had little effect on improving the air-conditioning performance, although the external nose morphologies including this feature could improve the transport of odorants to the superior olfactory slit in the upper nasal cavity. Thus, the unique external nose has little effect to improving the air-conditioning performance, and the impaired performance is more likely to be a consequence of modifications in the shape of the nasal cavity itself in *Homo* lineage since it was diversified from the other hominins, including australopithecines, in the beginning of the Early Pleistocene.

Inhaled air can be adjusted through the pharyngeal cavity to be fully conditioned in humans, even though it is not fully adjusted in the nasal cavity. In the phyletic divergence of *Homo* from the other hominin lineages, facial flattening and reorganization has reduced the dimensions of the horizontal oral cavity along with the nasal cavity and pushed the tongue down toward the pharynx, thereby lengthening the vertical pharyngeal cavity [8, 9]. Although the actual lengths of the pharyngeal cavity are unknown for each previous form of *Homo* [49, 50], the long pharyngeal cavity in extant humans contributes greatly to the sophisticated and flexible modifications of the topology of the supralaryngeal vocal tract from the glottis to the lips, which underlies human speech production [6, 51]. This feature is also believed to provide a disadvantage for the other physiological functions of the pharynx such as swallowing, increasing the risk of accidental aspiration during deglutition of food and liquid boluses [52–54]. However, the long pharyngeal cavity could in part compensate for the impaired air-conditioning performance within the short nasal cavity. The Late Pliocene to Early Pleistocene periods were characterized by a highly fluctuating climate and a gradual transition from warm and humid to cool and arid environments, especially in the northern hemisphere [13, 55]. These linked changes in the nasal and pharyngeal regions would in part have contributed to how flat-faced hominins, i.e., *Homo* members, must have survived such fluctuations in climate, before they moved “Out of Africa” in the Early Pleistocene to explore the more severe climates and ecological environments of Eurasia.

## Supporting Information

S1 Table Subjects, scans, and estimated parameters of respiration. abbreviations: MRI, magnetic resonance imaging; CT, Computed Tomography; CA, cross-sectional area at the oropharyngeal level; TV, estimated tidal volume; f, estimated frequency of breath; FV, flow velocity at the pharyngeal level. *CT scans of chimpanzees and macaques used here are deposited and released at the Digital Morphology Museum, KUPRI (dmm.pri.kyoto-u.ac.jp/archive/), under PRICT #.^†^estimate.
(DOCX)

S1 Fig Airflows and flow velocity in the nasal passage in humans. (A) Human volunteer 1; (B) human volunteer 3; (C) human volunteer 5; (D) human volunteer 6. The streamlines (upper) and contours (bottom) indicate the airflow direction and velocity distributions through the nasal passage, respectively. The streamline number reflects the relative airflow volume for a given subject.
(TIF)

S2 Fig Airflows and flow velocity in the nasal passage in nonhuman primates. (A) chimpanzee Mari; (B) chimpanzee Pendesa; (C) chimpanzee Reiko; (D) macaque Mff963; (D) macaque Mff1859; (F) macaque Mff2115; (G) macaque Mm1701; and (H) macaque Mm1715. The streamlines (upper) and contours (bottom) indicate the airflow direction and velocity distributions through the nasal passage, respectively. The streamline number reflects the relative airflow volume for a given subject.
(TIF)

S3 Fig Airflow and flow velocity in the nasal passage of the virtual topology models. (A, B) Human volunteer 1, (C, D) human volunteer 3, (E, F) human volunteer 5, (G, H) human volunteer 6. (A, C, E, G) No-valve model and (B, D, F, H) horizontal models. The streamlines (upper) and contours (bottom) indicate the airflow direction and velocity distributions through the nasal passage, respectively. The streamline number reflects the relative airflow volume for a given subject.
(TIF)
