# Supplementary material for: Impaired Air Conditioning within the Nasal Cavity in Flat-Faced Homo
Source: PLoS Comput Biol. 2016 Mar 24;12(3):e1004807. doi: 10.1371/journal.pcbi.1004807 (PMC4807068; doi:10.1371/journal.pcbi.1004807)
Supplement: S1 Table — abbreviations: MRI, magnetic resonance imaging; CT, Computed Tomography; CA, cross-sectional area at the oropharyngeal level; TV, estimated tidal volume; f, estimated frequency of breath; FV, flow velocity at the pharyngeal level. *CT scans of chimpanzees and macaques used here are deposited and released at the Digital Morphology Museum, KUPRI (dmm.pri.kyoto-u.ac.jp/archive/), under PRICT #.†estimate. (DOCX) [file pcbi.1004807.s001.docx]

**S1 Table. Subjects, scans, and estimated parameters of respiration.**

| **Species** | **Subjects** | **Sex** | **BW**  **(kg)** | **Age**  **(yrs)** | **Scans** | | | | **Mesh** | | **Respiration parameters** | | | |
| --- | --- | --- | --- | --- | --- | --- | --- | --- | --- | --- | --- | --- | --- | --- |
|  |  |  |  |  | **PRICT #^*^** | **Modality** | **Resolution**  **(mm/pixel)** | **Interval**  **(mm)** | **size**  **(**×**10^-4^)** | **Number (**×**10^6^)** | **CA**  **(mm^2^)** | **TV**  **(ml)** | **f**  **(Hz)** | **FV**  **(m/s)** |
| Humans,  *Homo sapiens* | Volunteer 1 | male | 70 | 36 |  | MRI | 0.375 | 0.75 | 3.40 | 3.28 | 134 | 638 | 0.278 | 2.65 |
|  | Volunteer 2 | male | 75 | 26 |  | MRI | 0.375 | 0.75 | 3.60 | 2.83 | 127 | 685 | 0.273 | 2.95 |
|  | Volunteer 3 | male | 70 | 28 |  | MRI | 0.375 | 0.75 | 3.00 | 2.91 | 119 | 638 | 0.278 | 2.98 |
|  | Volunteer 5 | female | 55 | 28 |  | MRI | 0.375 | 0.75 | 3.20 | 3.32 | 85 | 496 | 0.292 | 3.46 |
|  | Volunteer 6 | female | 58 | 29 |  | MRI | 0.375 | 0.75 | 3.50 | 3.40 | 179 | 525 | 0.292 | 1.71 |
| Chimpanzees,  *Pan troglodytes* | Mari | female | 44 | 35^†^ | 468 | CT | 0.249 | 0.20 | 2.70 | 3.56 | 133 | 394 | 0.314 | 1.86 |
|  | Pendesa | female | 40 | 33 | 371 | CT | 0.351 | 0.20 | 2.60 | 3.70 | 300 | 357 | 0.322 | 0.77 |
|  | Popo | female | 47 | 28 | 365 | CT | 0.390 | 0.20 | 2.50 | 3.30 | 274 | 422 | 0.309 | 0.95 |
|  | Reiko | female | 56 | 45^†^ | 457 | CT | 0.376 | 0.50 | 2.40 | 3.36 | 130 | 506 | 0.295 | 2.30 |
| Japanese macaques,  *Macaca fuscata* | Mff765 | female | 10.2 | 28 | 369 | CT | 0.225 | 0.20 | 2.10 | 3.25 | 74 | 86.1 | 0.459 | 1.070 |
|  | Mff963 | female | 6.6 | 26 | 374 | CT | 0.250 | 0.20 | 2.30 | 3.25 | 258 | 54.7 | 0.514 | 0.218 |
|  | Mff1859 | female | 8.7 | 9 | 46 | CT | 0.263 | 0.20 | 3.20 | 3.23 | 122 | 73.0 | 0.479 | 0.572 |
|  | Mff2115 | female | 7.9 | 5 | 380 | CT | 0.125 | 0.20 | 3.65 | 2.66 | 210 | 66.0 | 0.491 | 0.308 |
| Rhesus monkeys,  *Macaca mulatta* | Mm1701 | female | 6.7 | 6 | 394 | CT | 0.225 | 0.20 | 3.35 | 3.13 | 71 | 55.6 | 0.512 | 0.802 |
|  | Mm1715 | male | 7.1 | 6 | 395 | CT | 0.250 | 0.20 | 3.20 | 3.11 | 131 | 59.1 | 0.505 | 0.455 |

abbreviations: MRI, magnetic resonance imaging; CT, Computed Tomography; CA, cross-sectional area at the oropharyngeal level; TV, estimated tidal volume; f, estimated frequency of breath; FV, flow velocity at the pharyngeal level.

^*^CT scans of chimpanzees and macaques used here are deposited and released at the Digital Morphology Museum, KUPRI (dmm.pri.kyoto-u.ac.jp/archive/), under PRICT #.

^†^estimate
