# Supplementary figures and images for: Impaired Air Conditioning within the Nasal Cavity in Flat-Faced Homo
Source: PLoS Comput Biol. 2016 Mar 24;12(3):e1004807. doi: 10.1371/journal.pcbi.1004807 (PMC4807068; doi:10.1371/journal.pcbi.1004807)

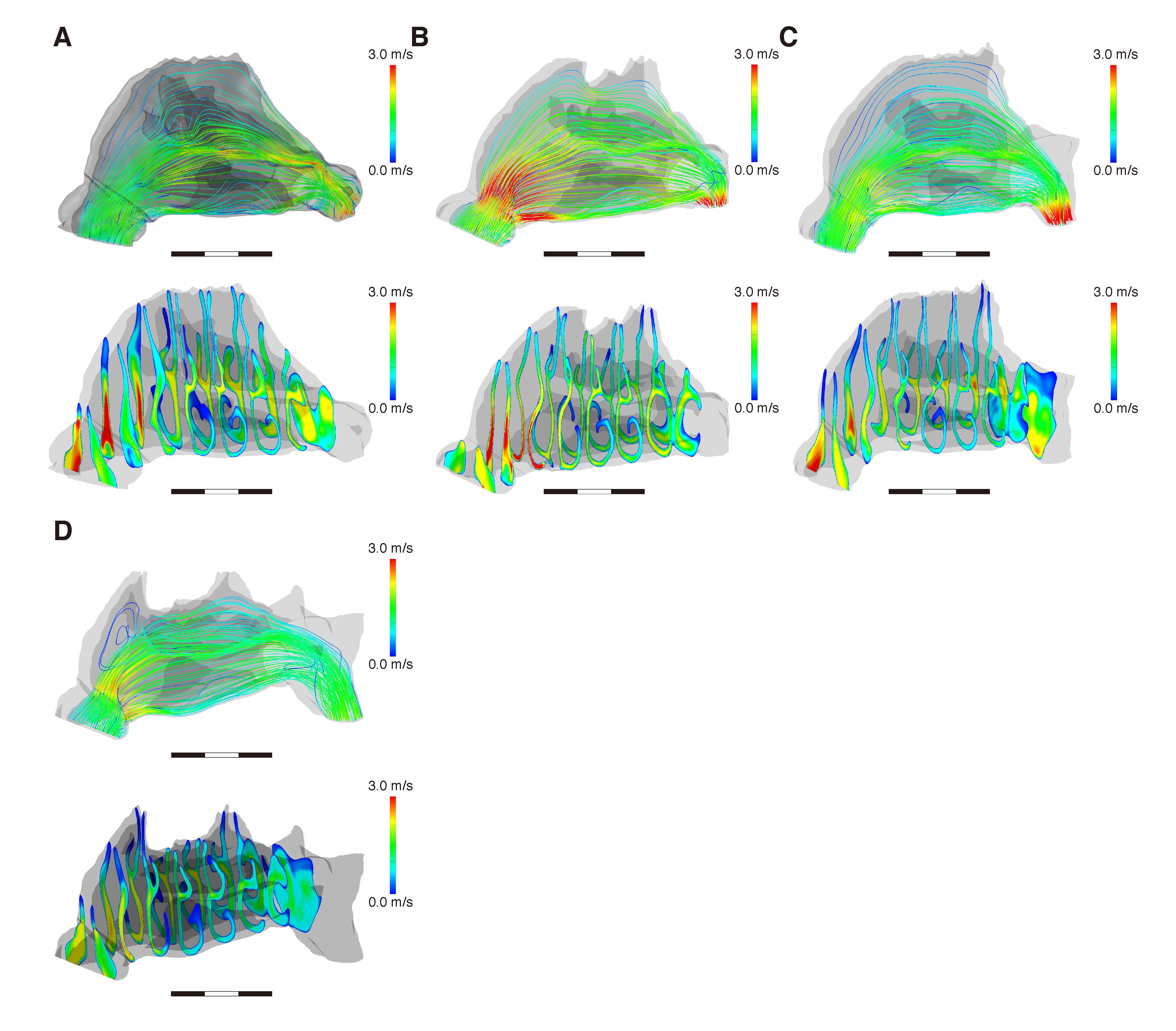

Supplement: S1 Fig — (A) Human volunteer 1; (B) human volunteer 3; (C) human volunteer 5; (D) human volunteer 6. The streamlines (upper) and contours (bottom) indicate the airflow direction and velocity distributions through the nasal passage, respectively. The streamline number reflects the relative airflow volume for a given subject. (TIF) [file pcbi.1004807.s002.tif]

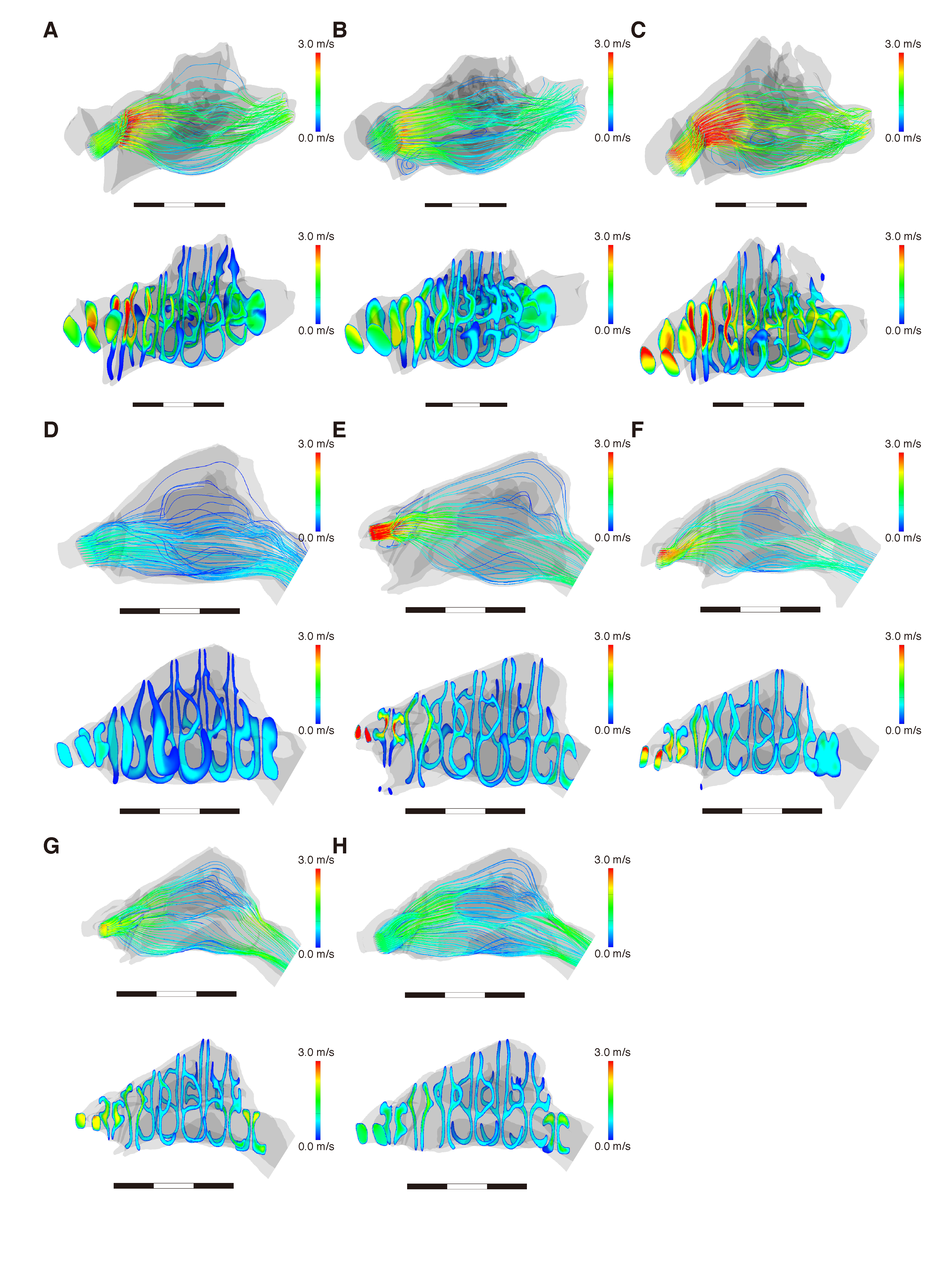

Supplement: S2 Fig — (A) chimpanzee Mari; (B) chimpanzee Pendesa; (C) chimpanzee Reiko; (D) macaque Mff963; (D) macaque Mff1859; (F) macaque Mff2115; (G) macaque Mm1701; and (H) macaque Mm1715. The streamlines (upper) and contours (bottom) indicate the airflow direction and velocity distributions through the nasal passage, respectively. The streamline number reflects the relative airflow volume for a given subject. (TIF) [file pcbi.1004807.s003.tif]

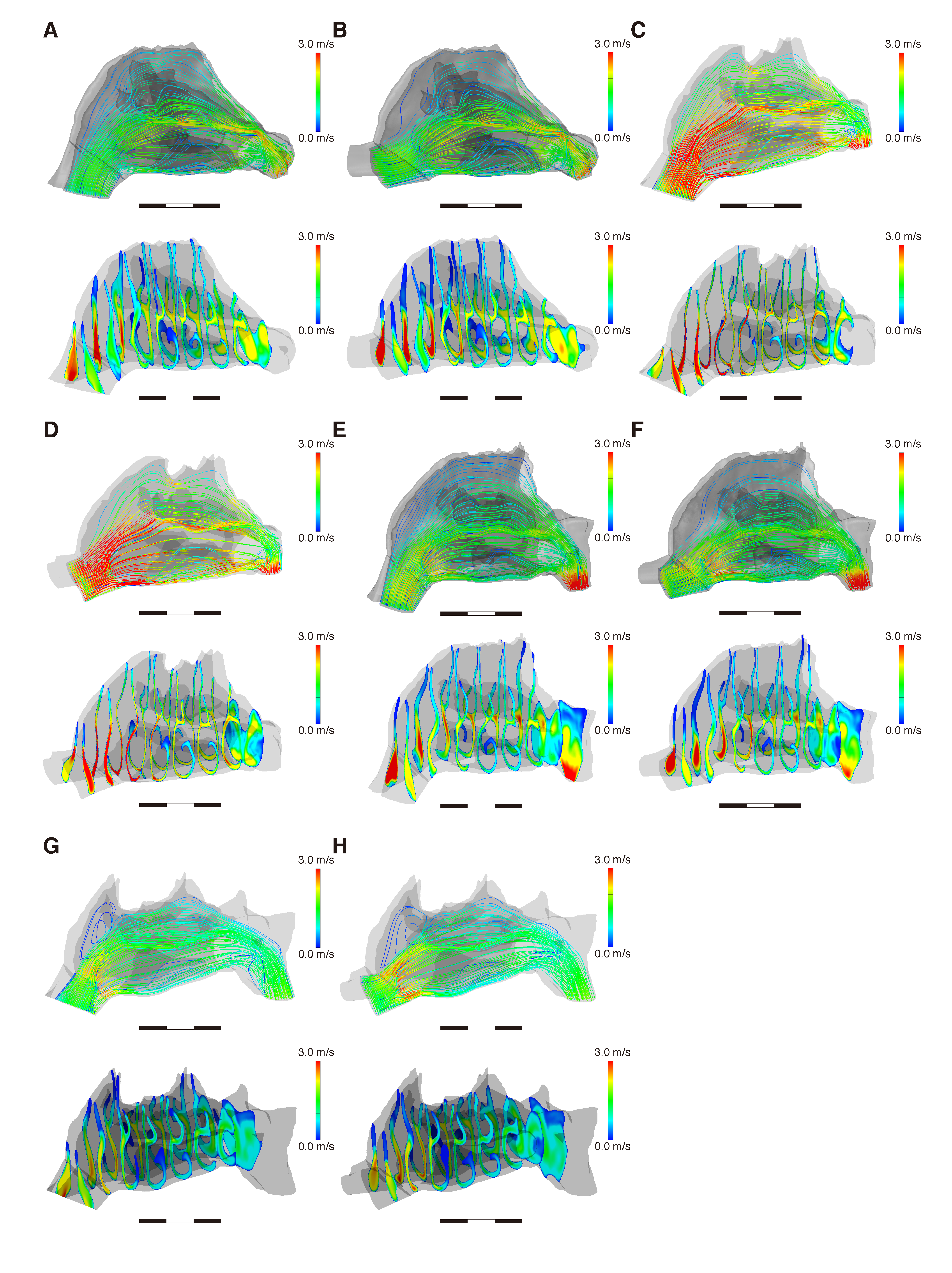

Supplement: S3 Fig — (A, B) Human volunteer 1, (C, D) human volunteer 3, (E, F) human volunteer 5, (G, H) human volunteer 6. (A, C, E, G) No-valve model and (B, D, F, H) horizontal models. The streamlines (upper) and contours (bottom) indicate the airflow direction and velocity distributions through the nasal passage, respectively. The streamline number reflects the relative airflow volume for a given subject. (TIF) [file pcbi.1004807.s004.tif]
